# Supplementary figures and images for: Chemo-omic pipeline enables discovery of prion synaptotoxic pathways and inhibitory drugs
Source: PLoS Pathog. 2026 Jun 18;22(6):e1014314. doi: 10.1371/journal.ppat.1014314 (PMC13278431; doi:10.1371/journal.ppat.1014314)

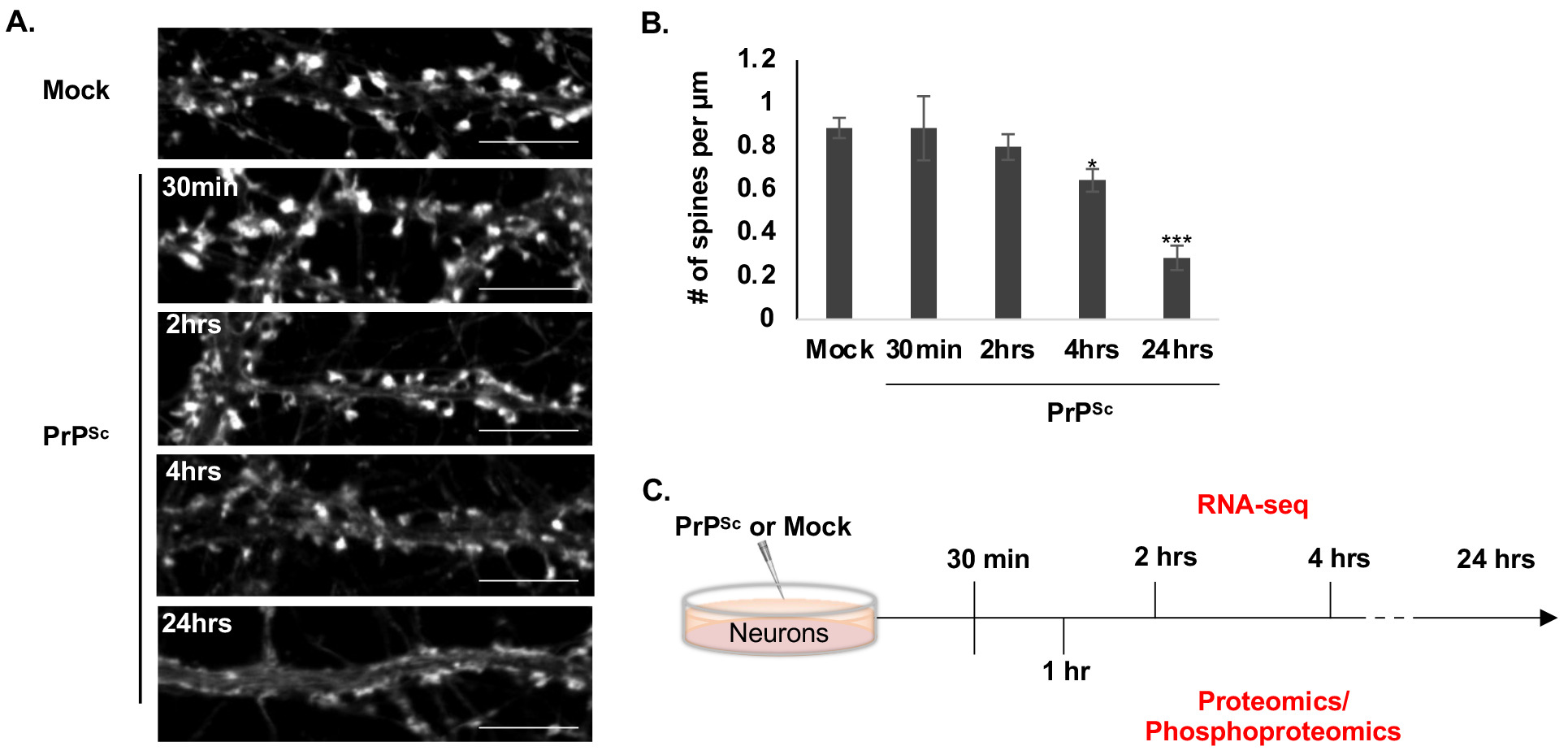

Supplement: S1 Fig — (A) Mature hippocampal neurons (21DIV) were treated with purified PrPSc for 30 min, 1 hour, 2 hours, 4 hours, or 24 hours, or with mock-purified material from uninfected brains for 24 hours. Neurons were then stained with fluorescent phalloidin to reveal dendritic spine morphology. Scale bars = 5 μm. (B) Quantification of spine number in PrPSc-treated neurons compared to mock-treated cultures. Pooled measurements were collected from 5-7 neurons and 15–25 dendritic regions from 3 independent experiments. Data are shown as the mean ± SEM. Statistical analysis was performed using unpaired t-tests. Significance is indicated as: *p < 0.05, ***p < 0.001. (C) Experimental set-up for RNA-seq and phosphoproteomic analysis. (TIF) [file ppat.1014314.s008.tif]

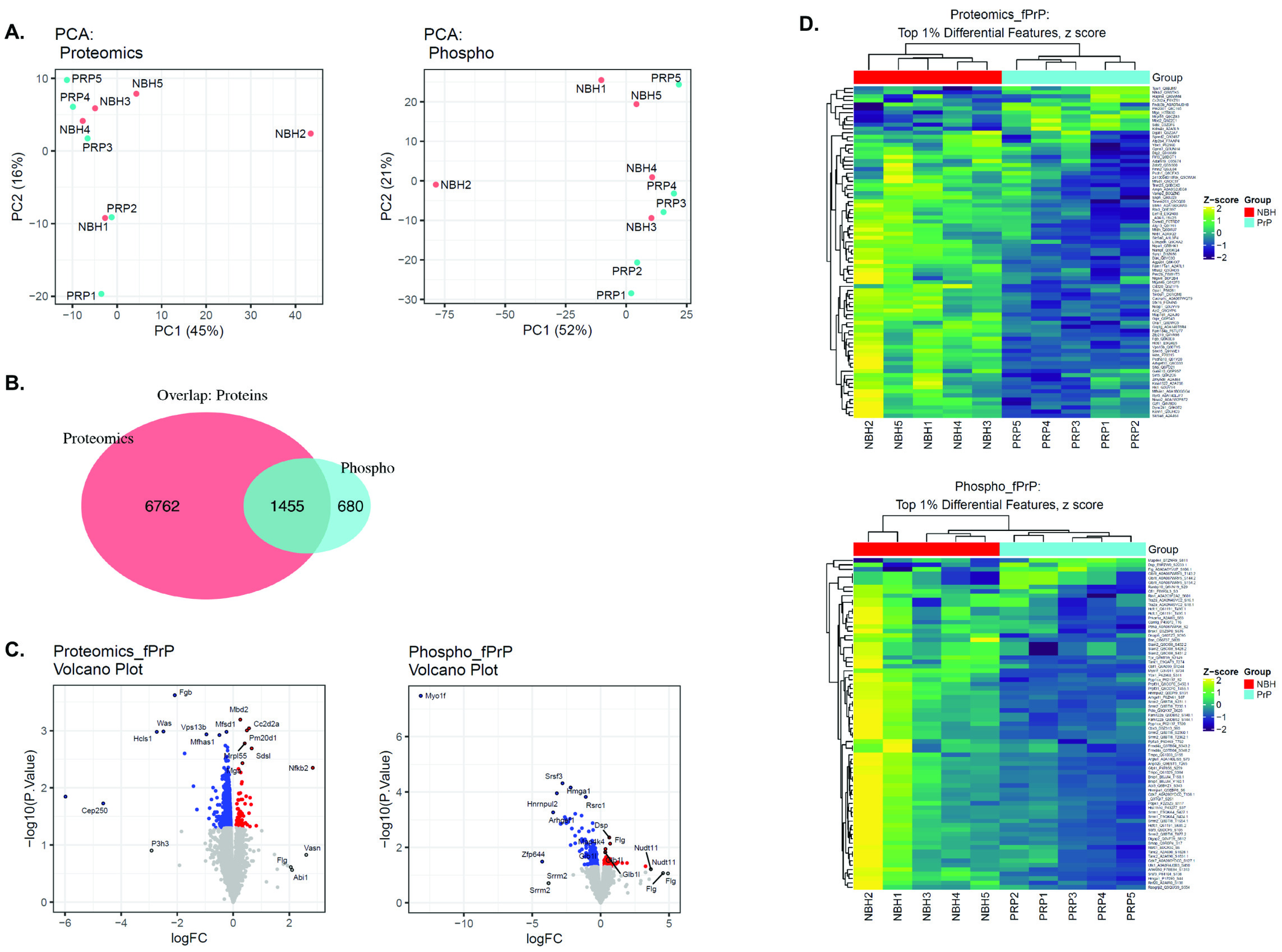

Supplement: S2 Fig — (A) Principal component analysis (PCA) of proteomic and phosphoproteomic data from five biological replicates of hippocampal neurons treated for 1 hr with PrPSc (PRP, cyan dots) or material that was mock-purified from uninfected brains (NBH, red dots). (B) Venn diagram comparing the number of proteins identified in the proteomic and phosphoproteomic analyses. The numbers refer to proteins that were identified based on at least two peptides. (C) Volcano plots depicting proteomic and phosphoproteomic data. Cut-offs are │log2fold-change│ > 0.25 and adjusted p-value <0.05. (D) Heat maps of proteomic and phosphoproteomic changes with Z-scores in the top 1% mapped onto corresponding genes. (TIF) [file ppat.1014314.s009.tif]

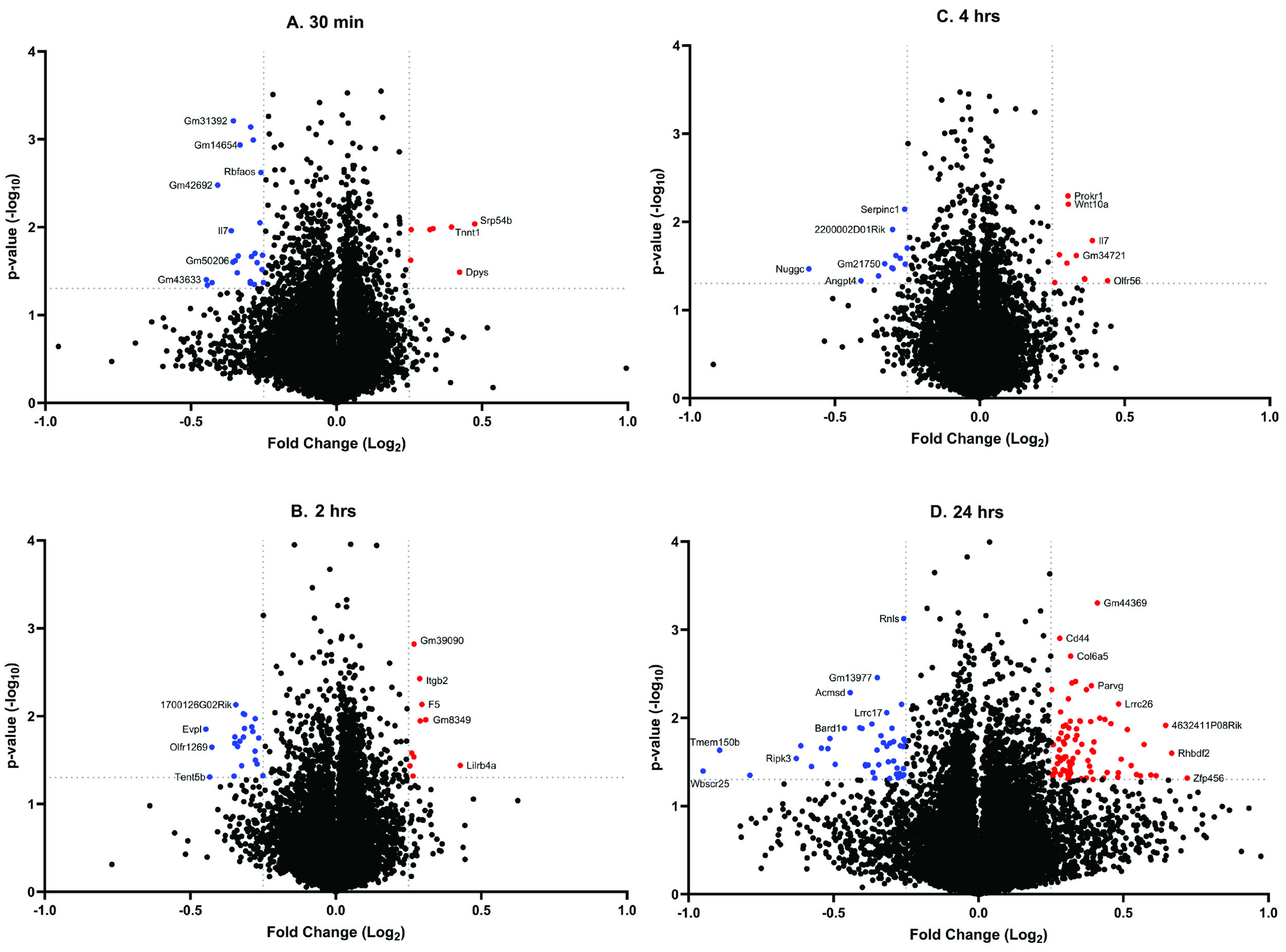

Supplement: S3 Fig — Neurons were treated with purified PrPSc for 30 min (A), 2 hr (B), 4 hr (C), and 24 hr (D). Cut-off values were |log2fold change│ > 0.25 and p-value <0.05. (TIF) [file ppat.1014314.s010.tif]

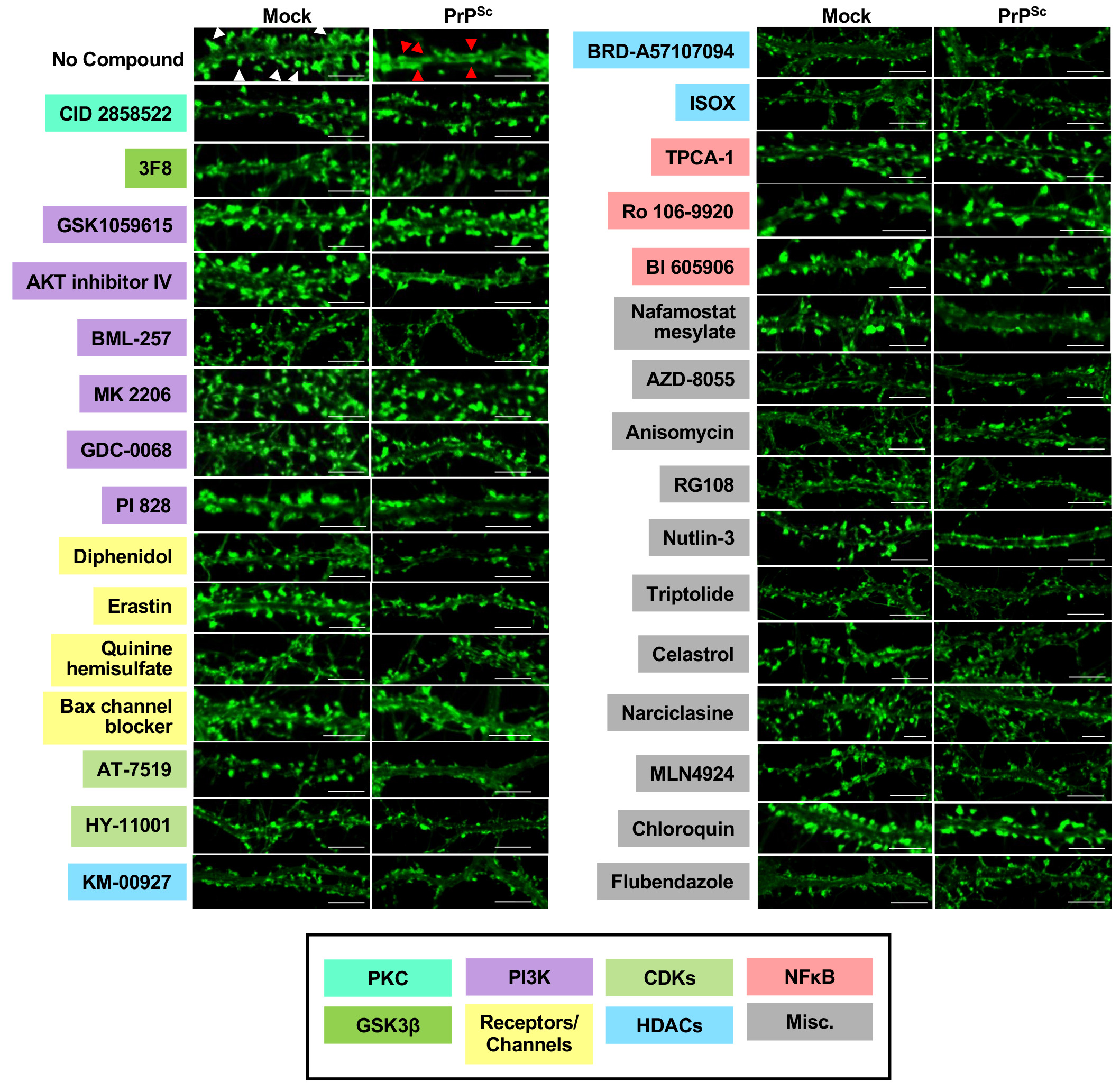

Supplement: S4 Fig — Hippocampal neurons were pretreated with non-toxic compounds listed in Table 1 at 250 nM, then exposed to PrPSc or mock-purified material for 24 hrs. After fixation, neurons were stained with Alexa 488-labeled phalloidin for visualization of dendritic spines. The top pair of panels shows neurons that were exposed to PrPSc or mock-purified material in the absence of any compound. White and red arrowheads in the top panels indicate healthy and retracted dendritic spines, respectively. Scale bars = 5 μm. Compounds and targets are color-coded as indicated in the legend and in Table 1. (TIF) [file ppat.1014314.s011.tif]

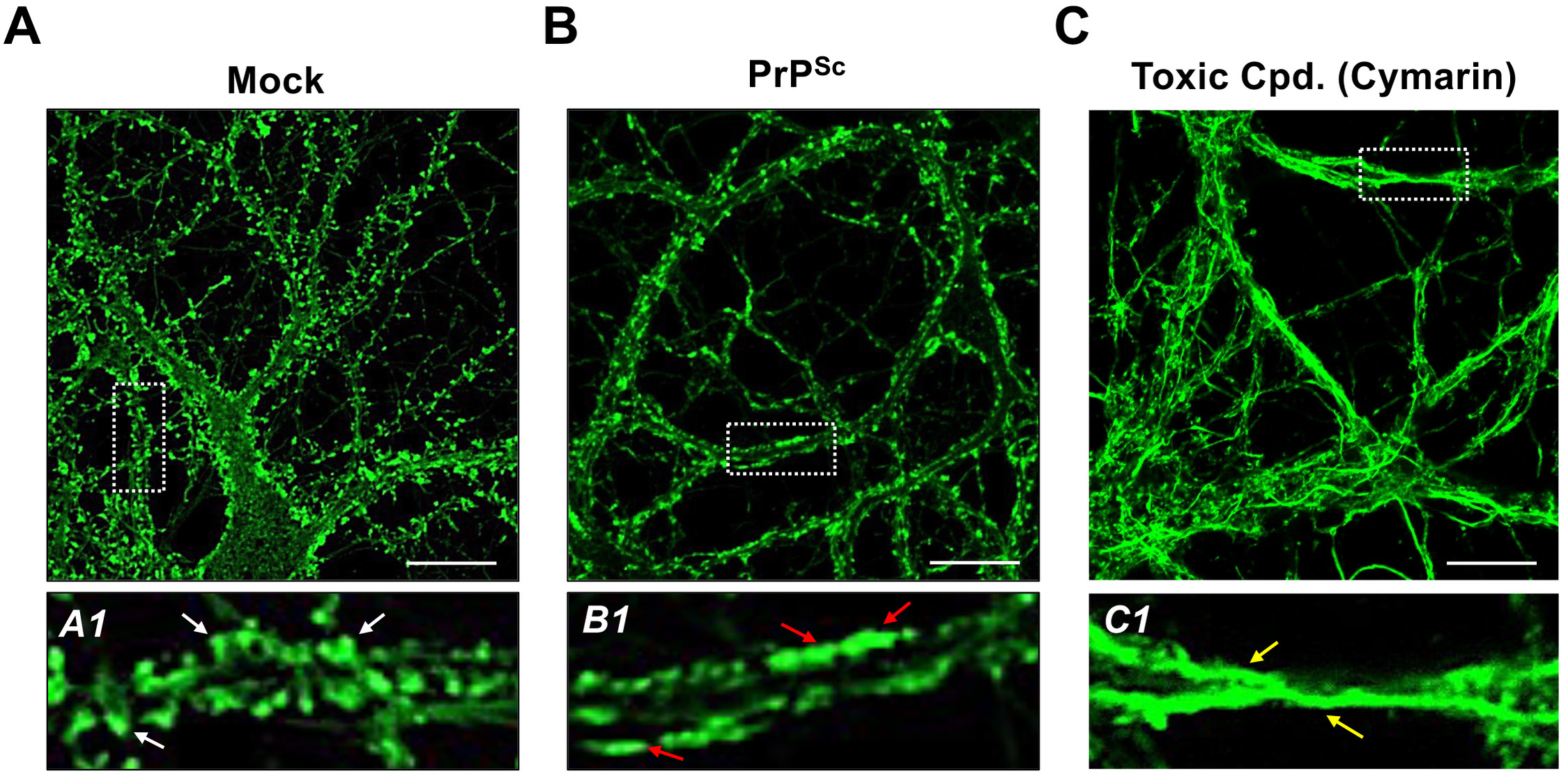

Supplement: S5 Fig — Hippocampal neuron cultures were treated with mock-purified material (A), with PrPSc (B), or with mock-purified material and one of the toxic compounds listed in Table 1 (Cymarin) at 250 nM (C). After 24 h, neurons were fixed and stained with AF488-phalloidin to visualize F-actin. Boxed regions in the square panels are shown at higher magnification in the corresponding rectangular panels below (A1-C1). White arrowheads (A1) indicate healthy spines, red arrowheads (B1) indicate retracted spines, and yellow arrowheads (C1) indicate damaged neurites containing actin bundles. Scale bars: 20 = μm. (TIF) [file ppat.1014314.s012.tif]

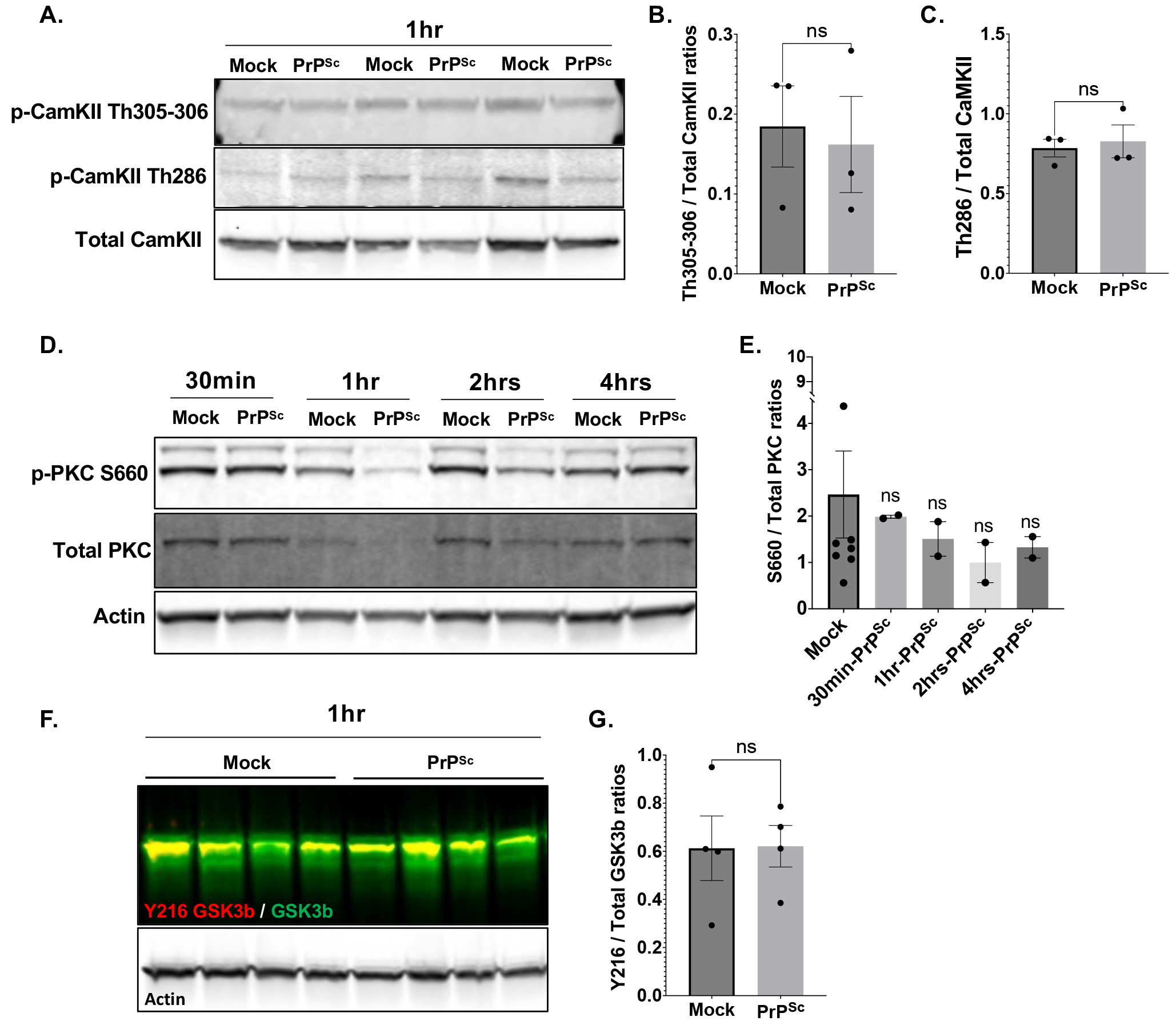

Supplement: S6 Fig — Western blots of neuronal lysates were probed with the indicated antibodies to total and phosphorylated forms of CaMKII (A), PKC (D), and GSK3β (F). Actin was used as a loading control in panels D and F. Blots were quantified by Image J (B, C, E, G). Data are shown as the mean ± SEM. Statistical analysis was performed using unpaired t-tests. Significance is indicated as: ns (not significant). (TIF) [file ppat.1014314.s013.tif]

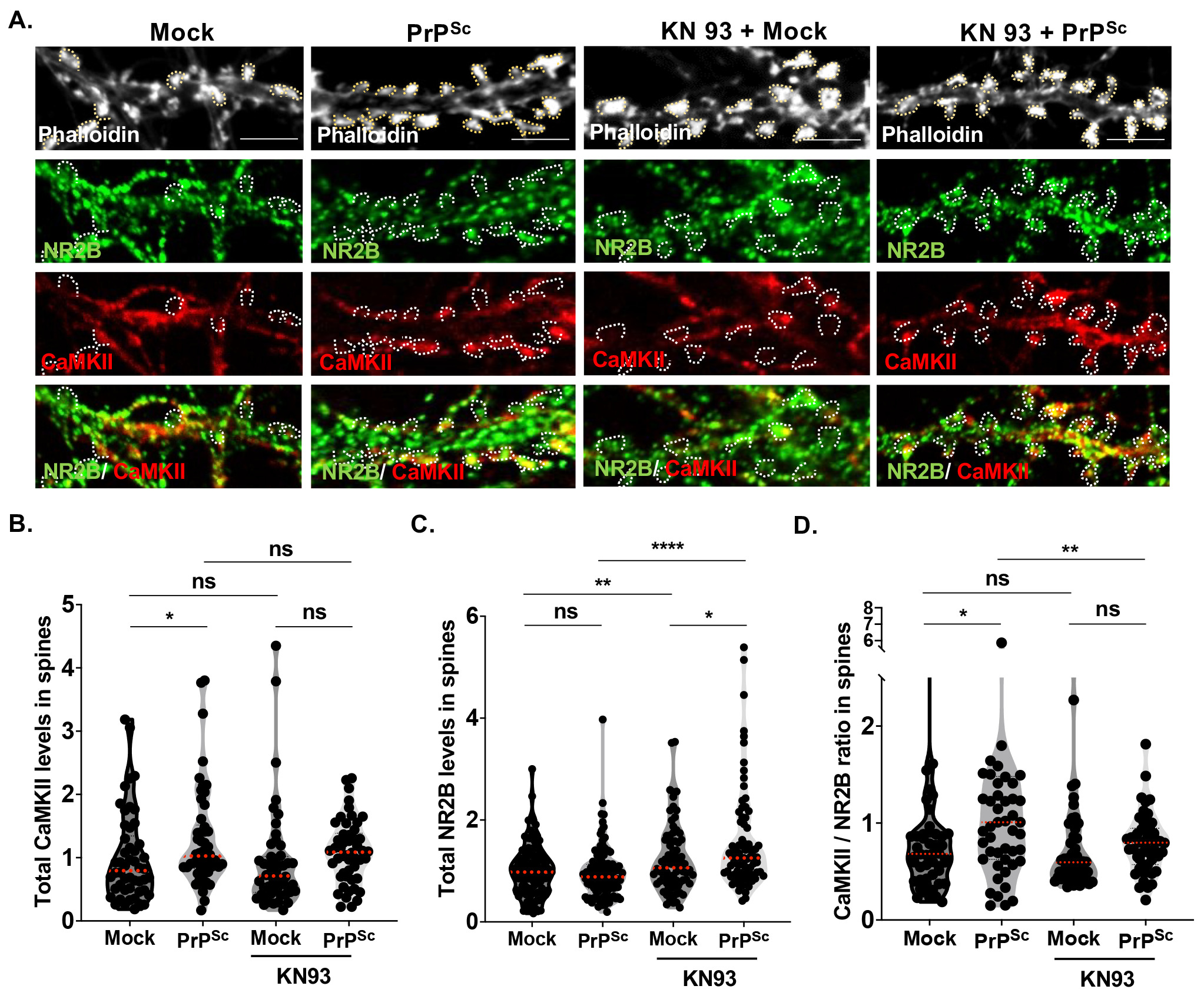

Supplement: S7 Fig — (A) Hippocampal neurons were pre-treated with KN-93 for 2 hours, then treated for 1 hour with either mock-purified material (Mock) or purified PrPSc (PrPSc). Cultures were then fixed and stained with fluorescent phalloidin (gray), along with antibodies to total CaMKII (red) and N-methyl D-aspartate receptor subtype 2B, NR2B (green). Dotted lines in the smaller panels outline the positions of intact spines, based on phalloidin staining. Scale bars = 5 μm. Violin plots show quantitation of Total CaMKII (B), Total NR2B (C), and CaMKII/NR2B (D) in spine regions. Measurements were collected from 5-7 neurons, and 15 dendritic regions from at least 2 independent experiments. Each data point for the PrPSc-treated samples was normalized to the average Mock value from the same experiment. Dotted red lines in each violin plot indicate the median. Statistical analysis was performed on SEM values using unpaired t-tests. Significance is indicated as: ns (not significant), *p < 0.05, **p < 0.01, ****p < 0.0001. The magnitude of the PrPSc-induced translocation of total CaMKII to spines in the control condition (no KN-93) was less than shown in Fig 4 (panels A, B, and E), reflecting variability between experiments. (TIF) [file ppat.1014314.s014.tif]

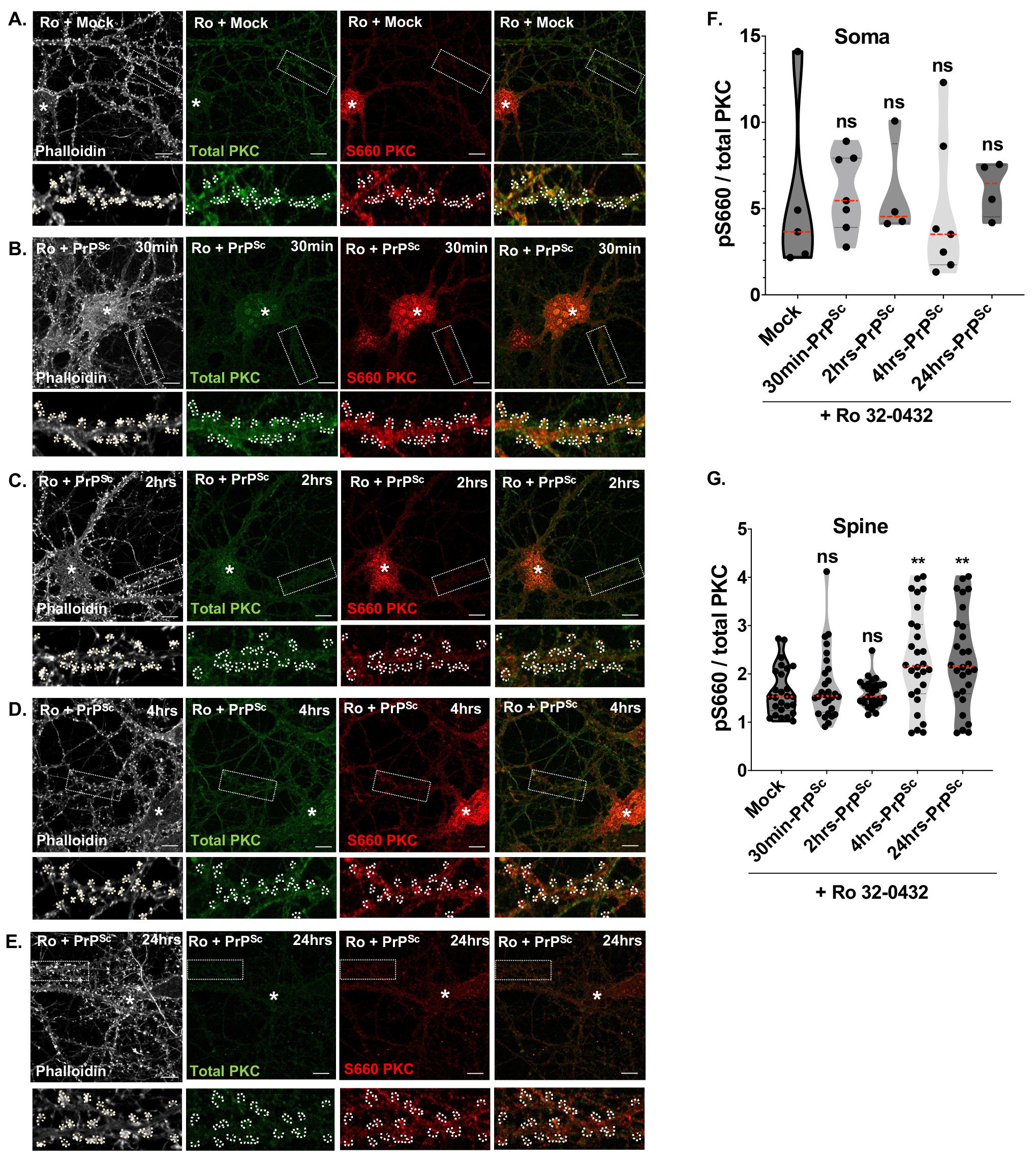

Supplement: S8 Fig — Hippocampal neurons were pre-treated with Ro 32–0432 hydrochloride for 2 hours and were then treated either with mock-purified material (A), or with purified PrPSc for 30 min (B), 2 hours (C), 4 hours (D) or 24 hours (E). After fixation, neurons were stained with fluorescent phalloidin (gray), along with antibodies to total PKC (green) and PKC-pS660 (red). Boxed regions in the square panels are shown at higher magnification in the rectangular panels below. Dotted lines in the higher magnification panels outline the positions of intact spines, based on phalloidin staining. Asterisks mark the locations of neuronal somata. Scale bars = 5 μm. Violin plots show quantitation of pS660/total PKC ratios within somata (F) and spines (G). Measurements were collected from 5-7 neurons, 30–45 dendritic and somatic regions from at least 2 independent experiments. Each data point for the PrPSc-treated samples was normalized to the average Mock value from the same experiment. Dotted red lines in each violin plot indicate the median. Statistical analysis was performed on SEM values using unpaired t-tests. Significance is indicated as: ns (not significant), **p < 0.01. (TIF) [file ppat.1014314.s015.tif]
