# Supplementary material for: Chemo-omic pipeline enables discovery of prion synaptotoxic pathways and inhibitory drugs
Source: PLoS Pathog. 2026 Jun 18;22(6):e1014314. doi: 10.1371/journal.ppat.1014314 (PMC13278431; doi:10.1371/journal.ppat.1014314)
Supplement: Attachment — Submitted filename: PLOS Patho 2026 rebutal final.docx [file ppat.1014314.s017.docx]

**Response to Reviewers**

We thank the reviewers for their careful evaluation of our manuscript entitled “Chemo-omic pipelines enable discovery of prion synaptotoxic pathways and inhibitory drugs.” We are grateful for the positive assessment of the novelty, rigor, and significance of our work. We have revised the manuscript to address all concerns and improve clarity. Below, we respond point-by-point.

**REVIEWER #1**

**Major issue 1:** Potential confounding by cytotoxicity

Reviewer comment: *“…a real risk that a cytotoxic effect may be misinterpreted as genuine synaptic protection. …Including viability assays is typically considered essential to support mechanistic interpretation of phenotypic results.”*

Response:

It seems unlikely that compounds that are intrinsically cytotoxic would preserve rather than degrade synaptic integrity, and thereby be misclassified as genuine (target-based) synaptic protectants. Nevertheless, several features of our experimental design and dataset argue against this possibility.

First, all compounds were tested in both PrP^Sc^-treated and mock-treated conditions, providing an internal control for each compound (see Fig. S4 and new Table S7). Compounds that are intrinsically toxic would be expected to reduce dendritic spine density and neuronal death in mock-treated neurons; however, this was not observed for the compounds identified as protective.

In addition, our assay incorporates morphological criteria that sensitively reveal neurotoxicity. Thus, compounds that produced clear signs of structural damage were readily detected and excluded from further analysis. Phalloidin staining of F-actin not only labels dendritic spines but also highlights toxicity-related cytoskeletal disruption, as documented in new Fig. S5. Toxic compounds consistently induced pronounced F-actin bundling, redistribution, and loss of discrete spine structures, accompanied by nuclear abnormalities detected by DAPI staining. Based on these features, twelve compounds were classified as toxic and removed from further consideration.

Importantly, synaptic stabilization and neurotoxicity represent biologically opposing outcomes, as neurotoxicity is typically associated with cytoskeletal collapse and spine loss rather than preservation of synaptic structure. Furthermore, our system captures early synaptotoxic events within a time window in which neuronal viability remains unaffected. Finally, the protective effects of selected compounds are supported by independent mechanistic readouts, including consistent effects on kinase localization. Together, these observations indicate that the effects we report reflect genuine synaptic protection rather than cytotoxic artifacts.

**Major Issue 2:** Semi-quantitative scoring.

Reviewer comment: *“… the scoring of the 52 compounds identified through the chemo-omics pipeline (Table 1 and Figure S4) appears to have been conducted in a semi-quantitative manner. However, the authors did not specify the criteria underlying the four classes used to categorize inhibition of spine retraction. Furthermore, the authors did not specify whether the robustness of scoring was underpinned by blinding and randomization.”*

Response:

The inhibitory effect of the compounds on spine retraction is shown on a semi-quantitative scale in Table 1 for simplicity in presentation. To improve transparency, we now provide the underlying quantitative data (spine counts) for the screened compounds in a new Table (Supplementary Table S7). Table S7 also includes compound treatments of Mock-treated cultures, which is relevant to our response to Major Issue 1 (above). We have also clarified in the Materials and Methods that image acquisition and analysis were performed in a blinded manner with randomized field selection to minimize bias. We wish to emphasize that all mechanistic conclusions are based on rigorous quantitative measurements of dendritic spine density using multiple independent inhibitors per target, and complementary biochemical and imaging approaches.

**Minor Issues:** Editorial and Data Presentation Modifications

Reviewer comment: *“… the authors may have had valid reasons for selecting the L1000 platform. …the limitations associated with the restricted set of directly measured genes should be acknowledged in the Discussion.”*

Response:

We acknowledge that the L1000 dataset directly measures only ~978 landmark genes, with the remainder of the transcriptome inferred computationally, which may limit gene-level resolution. In response to the reviewer’s prompting, we uncovered two studies published in 2022 (after we had commenced our analysis) from the group of Avi Ma’ayan in which deep learning was used to transform the L1000 signatures into RNA-seq-like, whole transcriptome datasets (Jeon *et al*., *BMC Bioinformatics*, 23:374; Evangelista *et al*., *Nucleic Acids Research,* Vol. 50: W697–W709). Although those studies claimed a high degree of concordance between the AI-derived and experimentally determined RNA-seq datasets, the former are nevertheless still imputed and not directly measured. Therefore, the gain in accuracy remains uncertain. In our study the L1000 database was used specifically for connectivity mapping rather than for primary gene expression analysis. Moreover, we used experimentally derived RNA-seq and phosphoproteomic datasets as inputs, together with functional validation of predicted compounds in primary neurons. In any case, we have now added a sentence to the Discussion acknowledging the limitation of the L1000 database.

**REVIEWER #2**

**Minor Issues 1:** *“… the rationale of why doing one timepoint for phosphoproteomics while assaying four time points for RNASeq analysis is unclear. This would need some explanation as it is important for similar studies by others.”*

Response:

The selection of a single early time point (1 hour) for phosphoproteomic analysis was based on our prior observations that PrP^Sc^ induces rapid, phosphorylation-dependent signaling events, including NMDAR-mediated Ca²⁺ influx and activation of kinases such as p38 MAPK within minutes to 1 hour of exposure. In contrast, transcriptional responses evolve more gradually, motivating the inclusion of multiple time points (0.5-24 hours) for RNA-seq to capture both early and delayed gene expression changes. We have described this rationale in the Results section (Time course of PrP^Sc^-induced synaptotoxicity).

**Minor Issues 2:** *“… explain why they test only one fixed drug concentration (250 nM) and for 24-hour treatment only. They could discuss whether this very rigorous approach could miss promising hits.”*

Response:

We agree that testing a single concentration and time point represents a simplification. A concentration of 250 nM for 24 hours was deliberately chosen to standardize the screening process. This concentration falls within a range commonly used for kinase inhibitors, and is sufficient to determine whether a compound is effective, has reduced efficacy, or causes toxicity, while minimizing off-target effects. The 24-hour time point corresponds to when we consistently observed PrP^Sc^-induced dendritic spine retraction, making it an optimal window to detect compound effects on synaptotoxicity. We acknowledge that this approach may miss compounds with distinct dose-response or temporal profiles and have added a statement to this effect in the Results section.

**Minor Issues 3:** “… *the Discussion part did not address whether performing phosphoproteomics or transcriptomics alone would have resulted in similar outcomes, and whether it really needs both approaches in this context. Another idea is whether combining drugs might provide additive effects while not increasing side effects. In general, this type of approach might be an ideal combination therapy for other anti-prion strategies brought forward.”*

Response:

While either transcriptomic or phosphoproteomic data alone can, in principle, be used for connectivity mapping, integrating both modalities provides complementary information; to our knowledge, ours is the first study to do this. We anticipated that phosphoproteomics would capture rapid, early signaling events immediately downstream of PrP^Sc^ exposure, whereas transcriptomics would reflect longer-term cellular responses. Importantly, prioritizing compounds supported by both datasets increased confidence in candidate selection and led to convergence on key kinases (CaMKII, PKC, and GSK3β). This integrative approach not only enhances robustness and predictive power compared to either modality alone, but also highlights potential opportunities for combination therapies, as targeting multiple nodes of the synaptotoxic network may provide additive benefits without increasing toxicity. We have now included these points in the Discussion section.

**Minor Issues 4:** “… *the treatment window appears very small with 4 hours, but the authors did not discuss how this relates to prion disease pathogenesis which goes over a long time and is not really synchronized.”*

Response:

Of course, prion and other neurodegenerative diseases are chronic conditions that evolve over extended periods of time, typically in mid- late-adult life. Our synaptotoxicity assay system is not meant to model all aspects of the disease, but rather to isolate a single, well established pathogenic feature (synaptic degeneration), which is recognized to be one of earliest changes observable in humans and animals. Prion synaptotoxicity in this model is easily scored and quantitated, and the cell-based nature of the system permits mechanistic dissection at the molecular level. In our *in vitro* system, 4 hours marks the earliest detectable dendritic spine retraction, while by 24 hours most spines have retracted, representing the maximal synaptotoxic effect. The time points for our analyses were chosen to capture both the onset and progression of spine loss. Early time points (0.5-2 hours) reveal rapid signaling and phosphorylation events that precede structural changes, 4 hours provides the first morphological readout, and 24 hours reflects the culmination of synaptotoxicity. This temporal mapping allows dissection of the signaling cascade and identification of key molecular targets, despite the chronic, asynchronous nature of prion disease *in vivo*. We have included additional discussion of these points in the Discussion section.

**Minor Point:** “*The authors mention on page 6 ‘minimal contamination from astrocytes’ (feeder layer). Could they put some numbers on this statement?”*

Response:

In our culture system, hippocampal neurons are prepared independently from astrocytes and plated on coverslips that are physically separated from the astrocyte feeder layer. Astrocytes remain confined to the underlying layer, without direct mixing with the neuronal population. Consistent with this, we rarely observe astrocytes in the neuronal layer based on staining (e.g., phalloidin labeling of neuronal structures). While we did not perform a formal quantification of astrocyte contamination in this study, these features of the culture system ensure that neuronal samples are highly enriched with minimal astrocytic contribution. We have clarified this point in the Results section.

**REVIEWER #3**

We have revised the manuscript to soften statements regarding prion propagation and kinase activation, clarified data interpretation where appropriate, and improved presentation of quantitative analyses, including justification of ratio-based measurements. We have also addressed specific concerns related to variability, figure interpretation, and terminology, and have incorporated additional clarifications in the text to ensure a more balanced and rigorous presentation of our findings.

**Minor Issues 1:** *“… too strong a statement in the abstract and introduction to say that the mechanism of prion propagation is well understood.”*

Response:

We have revised the Abstract, Author Summary, and Introduction to soften this statement and better reflect existing uncertainties regarding the structural basis of PrP^Sc^-PrP^C^ interactions and the conformational conversion process. Specifically, phrases such as “is now well understood” have been modified to more cautious language, while retaining the emphasis that the mechanisms of prion neurotoxicity remain less clearly defined.

**Minor Issues 2:** *“CaMKII: … I do not think the authors can support the claim that CaMKII was activated, based on the data presented. I suggest softening this statement.”*

Response:

We agree that the observed decrease in both pT286 (activating) and pT305/6 (inhibitory) phosphorylated CaMKII within the postsynaptic density (PSD) complicates a straightforward interpretation of kinase activation. Our data robustly show that total CaMKII rapidly translocates to dendritic spines following PrP^Sc^ exposure, accompanied by decreased inhibitory T305/6 phosphorylation, consistent with recruitment of the de-inhibited form of the enzyme. The reduction in pT286 may reflect rapid dephosphorylation by PSD-associated phosphatases, as suggested, but we acknowledge that this does not provide direct evidence of net activation. Accordingly, we have substituted more nuanced statements regarding CaMKII activation in the Results and Discussion sections to more accurately reflect the limitations of these data.

**Minor Issues 3:** *“For all CaMKII, PKC and GSK3B studies, a ratio of phosphorylated to total is presented, with some results showing large variability. Can the authors present the data for the experiments without the ratio? Does that change the interpretation? If only the ratio is shown, it needs to be better justified, as ratios can augment errors.”*

Response:

Normalizing phosphorylated kinase levels to total kinase protein, which is standard in the field, is essential for accurately interpreting kinase activity, as absolute phospho levels alone do not account for differences in protein abundance across samples. Importantly, we have examined the raw phospho signals without normalization, and they display the same trends and similar variability as the ratios, indicating that the variability reflects the natural heterogeneity of phosphorylation rather than an artifact of ratio calculation. Thus, using the ratio allows robust interpretation while faithfully representing the biological signal.

**Minor Issues 4:** *“… Stating that KN93 prevents translocation to the spines based on this figure seems like an overstatement. Panel B, PrPSc with vs without KN93 does not show any statistical difference. Suggest softening statement / figure title.”*

Response:

The PrP^Sc^-induced translocation of total CaMKII to spines was consistent across several experiments, although the magnitude of the effect varied. For example, the experiment shown in Fig. 5 (panels A, B, and E) revealed more dramatic translocation in comparison to the experiment shown in Fig. S7 (Previously S6: panels A and B), where the effect was more modest. This variability reflects the inherent limitations of quantitating immunofluorescence signals emanating from multiple dendritic spines from a population of primary neurons. However, given this limitation, Fig. S7 does show that the small, but statistically significant increase in total CaMKII in spines caused by PrP^Sc^ was abolished by KN93. We have substituted a less definitive title for the Fig. S7 legend, and have noted the issue of experimental variability in the body of the legend.

**Minor Issues 5:** “*Fig. S7 - in the Spines, at 30min it seems there is more variability, which is lost by 2hrs, and then increases again at 4 and 24 hrs. Can the authors comment on this? It isn’t so clear cut as to say that PS660/total PKC just increases at 4 and 24 hours, as it would appear that the majority of the data stays within the range of mock, with enough outliers to pull the average into significance. Is this another issue from using a ratio instead of just total pS660? Do the authors get the same results without using the ratio? Is the variability better or worse?*”

Response:

The higher variability observed at 30 minutes likely reflects an early adaptive phase following PrP^Sc^ exposure, during which signaling changes are more heterogeneous across dendritic spines. By 2 hours, this variability decreases, consistent with a more stabilized signaling state. At 4 and 24 hours, we observe a clearer increase in pS660 phosphorylation, although some variability remains, possibly representing subsequent regulatory phases during the progression of synaptotoxicity. Importantly, we confirmed that the same temporal trends and variability are present whether analyzing the ratio of pS660 to total PKC or the absolute pS660 levels, indicating that the use of ratios does not artificially inflate variability but rather accounts for fluctuations in total protein levels. Note: the figure in question is now S8.

**Minor Issues 6:** *“In Table S6, antibodies Alexa “Flour” should read “Fluor””*

Response:

“Alexa Flour” has been corrected to “Alexa Fluor” in Table S6.

**Minor Issues 7:** *“… Was any attempt made to determine whether morphologies were affected by PrPSc exposure or the various inhibitors? The authors might consider commenting on spine morphologies (assuming these are present in this culture system).”*

Response:

While dendritic spines in our culture system are morphologically diverse, as they are *in vivo*, our analysis focused on overall spine density as a robust and quantifiable readout of synaptotoxicity. In this system, PrP^Sc^ exposure is associated with characteristic spine flattening and retraction, which we use as morphological indicators of synaptic damage. However, we did not perform a systematic classification of spine subtypes in response to PrP^Sc^ or inhibitor treatments. We agree that a more detailed analysis of spine morphology could provide additional insights and represents an interesting direction for future studies.

**Minor Issues 8:** “*GSK3B: … the logic that this inhibition therefore predicts that GSK3B would auto-phosphorylate itself…. If inhibitors are used, does phosphorylation no longer occur?*”

Response:

We agree with the reviewer that our original wording was unclear. Our intention was not to imply that inhibition of GSK3β predicts its autophosphorylation. Rather, based on the known biology of GSK3β, in which phosphorylation at Y216 is associated with its active state and can occur through autophosphorylation, we examined whether PrP^Sc^ treatment alters this modification. We found that PrP^Sc^ increases the level of GSK3β-pY216 in dendritic spines, consistent with activation of the kinase. The use of GSK3β inhibitors demonstrates that kinase activity is functionally required for spine retraction but does not directly inform on the mechanism of Y216 phosphorylation. We have revised the text in the appropriate section of the Results to clarify this point and to avoid implying a causal relationship between inhibitor effects and autophosphorylation.
