# Supplementary material for: Chemo-omic pipeline enables discovery of prion synaptotoxic pathways and inhibitory drugs
Source: PLoS Pathog. 2026 Jun 18;22(6):e1014314. doi: 10.1371/journal.ppat.1014314 (PMC13278431; doi:10.1371/journal.ppat.1014314)
Supplement: Peer review history

# Peer review: Decision Letter 0

**DOI:** 10.1371/journal.ppat.1014314.r001

**Authors:** Surachai Supattapone

27 Mar 2026

-->PPATHOGENS-D-26-00293

Chemo-omic pipeline enables discovery of prion synaptotoxic pathways and inhibitory drugs

PLOS Pathogens

Dear Dr. Harris,

Thank you for submitting your manuscript to PLOS Pathogens. After careful consideration, we feel that it has merit but does not fully meet PLOS Pathogens's publication criteria as it currently stands. Therefore, we invite you to submit a revised version of the manuscript that addresses the points raised during the review process. The reviewers thought that your study was significant and important, using cutting edge technology to identify and validate potential kinases involved in prion-induced dendritic spine retraction, suggesting new targets for prion therapeutics. The decision of Major Revision was based on the lack of viability (toxicity) data for the inhibitors tested, raising concerns that a cytotoxic effect may be misinterpreted as genuine synaptic protection. Additional concerns about the methods, including the criteria underlying the four classes used to categorize inhibition of spine retraction and the limited drug concentration and treatment times tested, also need to be addressed.

Finally, while the data were considered novel and mechanistically relevant, there were some questions about data interpretation and whether some of the conclusions/statements were justified by the data. Please be sure to address all of these issues in your response to the reviewers.

Please submit your revised manuscript by May 26 2026 11:59PM. If you will need more time than this to complete your revisions, please reply to this message or contact the journal office at plospathogens@plos.org. When you're ready to submit your revision, log on to https://www.editorialmanager.com/ppathogens/ and select the 'Submissions Needing Revision' folder to locate your manuscript file.

Please include the following items when submitting your revised manuscript:

* A letter that responds to each point raised by the editor and reviewer(s). You should upload this letter as a separate file labeled 'Response to Reviewers'. This file does not need to include responses to any formatting updates and technical items listed in the 'Journal Requirements' section below.

* A marked-up copy of your manuscript that highlights changes made to the original version. You should upload this as a separate file labeled 'Revised Manuscript with Track Changes'.

* An unmarked version of your revised paper without tracked changes. You should upload this as a separate file labeled 'Manuscript'.

If you would like to make changes to your financial disclosure, competing interests statement, or data availability statement, please make these updates within the submission form at the time of resubmission. Guidelines for resubmitting your figure files are available below the reviewer comments at the end of this letter.

We look forward to receiving your revised manuscript.

Kind regards,

Suzette A. Priola

Academic Editor

PLOS Pathogens

Surachai Supattapone

Section Editor

PLOS Pathogens

Sumita Bhaduri-McIntosh

Editor-in-Chief

PLOS Pathogens

orcid.org/0000-0003-2946-9497

Michael Malim

Editor-in-Chief

PLOS Pathogens

orcid.org/0000-0002-7699-2064

**Journal Requirements:**

1) Please ensure that the CRediT author contributions listed for every co-author are completed accurately and in full.

At this stage, the following Authors/Authors require contributions: Nhat T.T. Le, Robert C.C. Mercer, Cheng Fang, Aravind Sundaravadivelu, Adam T. Labadorf, WeiWei Lin, Julian Kwan, Benjamin Blum, Andrew Emili, and David A. Harris. Please ensure that the full contributions of each author are acknowledged in the "Add/Edit/Remove Authors" section of our submission form.

The list of CRediT author contributions may be found here: https://journals.plos.org/plospathogens/s/authorship#loc-author-contributions

2) Please upload all main figures as separate Figure files in .tif or .eps format. For more information about how to convert and format your figure files please see our guidelines:

https://journals.plos.org/plospathogens/s/figures

3) Please amend your detailed Financial Disclosure statement. This is published with the article. It must therefore be completed in full sentences and contain the exact wording you wish to be published.

State what role the funders took in the study. If the funders had no role in your study, please state: "The funders had no role in study design, data collection and analysis, decision to publish, or preparation of the manuscript.".

If you did not receive any funding for this study, please simply state: u201cThe authors received no specific funding for this work.u201d

**Reviewers' Comments:**

Reviewer's Responses to Questions

**Part I - Summary**

Please use this section to discuss strengths/weaknesses of study, novelty/significance, general execution and scholarship.

Reviewer #1: In their manuscript, “Chemo-omic pipelines enables discovery of prion synaptotoxic pathways and inhibitory drugs”, Le et al. aim to identify druggable targets associated with prion-induced dendritic spine retraction. To this end, the authors analysed transcriptomic and proteomic changes in primary hippocampal neurons exposed to PrPSc. They subsequently interrogated the resulting PrPSc-evoked expression signatures against drug-induced expression profiles derived from human cell lines, available in the public databases L1000 and P100. Based on the premise that perturbations producing similar expression profiles may share common targets or mechanisms of action, the authors instead focussed on perturbations that generated opposing gene expression signatures in order to identify potentially antagonistic effects. This analysis led to the identification of 17 chemical compounds capable of preventing dendritic spine retraction, and their respective targets (Table 1). Focusing on the protein kinases CaMKII, PKC and GSK3ß, the authors further investigated their role in PrPSc-induced spine retraction. While no evidence for changes in phosphorylation was observed (Fig. S5), the authors report alterations in the translocation and/or accumulation of CaMKII, PKC and GSK3ß to postsynaptic densities and dendritic spines, respectively (Fig. 5-7), supporting a potential functional role for these kinases in mediating PrPSc-induced dendritic spine retraction.

This study leverages a thoroughly validated primary neuronal model of prion-induced dendritic spine retraction to identify toxicity-associated cellular targets and corresponding small-molecule inhibitors. The Harris group has substantially advanced neuronal in vitro systems to investigate how prions compromise synaptic function and ultimately lead to synaptic loss. The authors here employed a cutting-edge computational approach to identify druggable targets by interrogating reference databases containing transcriptional profiles of human cell lines treated with small molecules, an experimental framework originally developed at the Broad Institute. By comparing PrPSc-induced gene and phospho-protein expression profiles with reference signatures generated by chemical or genetic perturbations, this strategy enables inference of drug mechanisms, gene targets, and candidate therapeutic compounds.

To my knowledge, this is the first report applying computational analysis of gene expression profiles against public databases in the field of prion diseases, addressing a key limitation of conventional RNA sequencing studies: the identification of gene targets associated with a given phenotype. Expression signature-based pipelines are amenable to generative AI-supported analyses and may link diseases with the genes that cause them and drugs that could treat them more effectively.

Reviewer #2: The manuscript by Nhat T.T. Lee and colleagues describes a very interesting chemo-omic pipeline approach that enabled the discovery of drugs and drug classes that inhibit early synaptotoxicity events induced by prions/PrPSc. Using prion exposure to hippocampal primary neurons as experimental model for synaptotoxic signaling, the authors first characterized the very early changes in both phosphoproteome and transcriptome associated with dendritic spine retraction. They then use this data to query available drug signature databases, looking for inverse signatures that could reverse the events they found in their -omics screens. This approach identified three major protein kinase targets. The authors very convincingly validated these pathways using the identified lead drugs, describing a signaling network that mediates prion-induced synaptotoxicity.

The manuscript describes a variety of very important data and provides novel mechanistic insights. First, the authors describe the very early events that underlie prion toxicity, an area very underdeveloped in prion research and pioneered largely by this group. Second, the concept of querying chemo-omic pipelines for inverse signatures is both audacious and compelling. Third, the authors used a very robust and elegant biological model to thoroughly validate their hits. The authors provide an impressive amount of data that is novel and mechanistically relevant. An interesting overall finding is that transcriptomic signature changes were very modest in this context, and that the very early pathological changes depend more on phosphorylation events and changes in subcellular localization.

Taken together, the authors provide very important data which are novel and significant. The manuscript is well done, experiments are clearly described, and conclusions are justified by the experimental data.

Reviewer #3: The authors make use of a primary neuronal cell culture assay (layered over astrocytes) to explore the earliest pathological changes to dendritic spines after exposure to purified RML strain prions. Using transcriptomic and phosphoproteomic profiles of prion-exposed neurons, combined with a small-molecule discovery pipeline, they identified inhibitors of CaMKII, PKC and GSK3β that prevented dendritic spine loss. They then used these to examine the timeline of phosphorylation and cellular localization of CaMKII, PKC and GSK3β, leading them to propose a timeline and mechanism for dendritic spine loss in prion infection. They provide evidence that the earliest response of neurons to prion exposure is at the level of protein phosphorylation-dependent signaling, before there is any significant changes in gene expression.This is a significant contribution to the prion field and will likely stimulate further research into pathogenesis and treatment based on these pathways.

The data presented is satisfactory for the most part. Some conclusions / statements should be softened or better justified.

**********

**Part II – Major Issues: Key Experiments Required for Acceptance**

Please use this section to detail the key new experiments or modifications of existing experiments that should be absolutely required to validate study conclusions.

Generally, there should be no more than 3 such required experiments or major modifications for a "Major Revision" recommendation. If more than 3 experiments are necessary to validate the study conclusions, then you are encouraged to recommend "Reject".

Reviewer #1: 1. The authors monitor fluorescence changes of fluorochrome-conjugated filamentous actin to quantify dendritic spine retraction as a measure for synaptotoxicity. However, since the study assesses rescue of spine retraction as the primary endpoint, there is a real risk that a cytotoxic effect may be misinterpreted as genuine synaptic protection. While the authors report toxicity for some of the small inhibitors in Table 1, I did not find viability (toxicity) data for the inhibitors tested in this manuscript, nor a methods section on quantification of cell viabilities. Including viability assays is typically considered essential to support mechanistic interpretation of phenotypic results.

2. The effects of CaMKII, PKC, and GSK3β inhibition on dendritic spine density were quantitatively assessed in Figure 4 according to the “dendritic spine quantitation” protocol described in the Methods section (page 26). In contrast, the scoring of the 52 compounds identified through the chemo-omics pipeline (Table 1 and Figure S4) appears to have been conducted in a semi-quantitative manner. However, the authors did not specify the criteria underlying the four classes used to categorize inhibition of spine retraction. Furthermore, the authors did not specify whether the robustness of scoring was underpinned by blinding and randomization.

Reviewer #2: none

Reviewer #3: I do not think any new experiments are required.

**********

**Part III – Minor Issues: Editorial and Data Presentation Modifications**

Please use this section for editorial suggestions as well as relatively minor modifications of existing data that would enhance clarity.

Reviewer #1: 1. The L1000 dataset measures expression changes for approximately 978 landmark genes, while the expression levels of the remaining ca. 22,000 genes are computationally inferred (Duan et al., 2014; doi:10.1093/nar/gku476). Although subsequent developments have expanded coverage towards near genome-wide representation (Alpern et al., 2019; doi:10.1186/s13059-019-1671-x), the authors may have had valid reasons for selecting the L1000 platform. This choice should therefore not be viewed as a weakness of the study; however, the limitations associated with the restricted set of directly measured genes should be acknowledged in the Discussion.

Reviewer #2: There are only a few minor issues the authors could address.

First, the rationale of why doing one timepoint for phosphoproteomics while assaying four time points for RNASeq analysis is unclear. This would need some explanation as it is important for similar studies by others. Second, the authors should explain why they test only one fixed drug concentration (250 nM) and for 24-hour treatment only. They could discuss whether this very rigorous approach could miss promising hits. Third, the Discussion part did not address whether performing phosphoproteomics or transcriptomics alone would have resulted in similar outcomes, and whether it really needs both approaches in this context. Another idea is whether combining drugs might provide additive effects while not increasing side effects. In general, this type of approach might be an ideal combination therapy for other anti-prion strategies brought forward. Finally, the treatment window appears very small with 4 hours, but the authors did not discuss how this relates to prion disease pathogenesis which goes over a long time and is not really synchronized.

Minor point:

1) The authors mention on page 6 ‘minimal contamination from astrocytes’ (feeder layer). Could they put some numbers on this statement?

Reviewer #3: 1) I think it is too strong a statement in the abstract and introduction to say that the mechanism of prion propagation is well understood. I would argue that we do not actually know how PrPSc, or which form of PrPSc, interacts with PrPC and how the conversion process takes place structurally. Fibrils structures are being solved, but it is not clear that these structures necessarily represent the initial formed structures. All this to say, I would soften the statement.

2) CaMKII: When western blots did not corroborate the changes is kinase phosphorylation, they looked at cellular distribution and found that CaMKII translocates to dendritic spines soon after exposure to PrPSc. However, in the dendritic spines, there was a decrease in the amount of T305/6 phosphorylated CaMKII (the inhibited form) AND a decrease in the pT286 phosphorylated CaMKII (the activated site). The authors speculate that the reduction of pT286 form is due to rapid dephosphorylation by PSD-associated phosphatases. If so, would this not affect both T305/6 and pT286 forms, making it difficult to conclude which, if either, was overly phosphorylated? I do not think the authors can support the claim that CaMKII was activated, based on the data presented. I suggest softening this statement.

3) For all CaMKII, PKC and GSK3B studies, a ratio of phosphorylated to total is presented, with some results showing large variability. Can the authors present the data for the experiments without the ratio? Does that change the interpretation? If only the ratio is shown, it needs to be better justified, as ratios can augment errors.

4) Figure S6 the difference between total CaMKII levels between Mock and PrPSc with or without KN93 seems modest at best. Stating that KN93 prevents translocation to the spines based on this figure seems like an overstatement. Panel B, PrPSc with vs without KN93 does not show any statistical difference. Suggest softening statement / figure title.

5) Fig S7 – in the Spines, at 30min it seems there is more variability, which is lost by 2hrs, and then increases again at 4 and 24 hrs. Can the authors comment on this? It isn’t so clear cut as to say that PS660/total PKC just increases at 4 and 24 hours, as it would appear that the majority of the data stays within the range of mock, with enough outliers to pull the average into significance. Is this another issue from using a ratio instead of just total pS660? Do the authors get the same results without using the ratio? Is the variability better or worse?

6) In Table S6, antibodies Alexa “Flour” should read “Fluor”.

7) Dendritic spines come in different morphologies. Was any attempt made to determine whether morphologies were affected by PrPSc exposure or the various inhibitors? The authors might consider commenting on spine morphologies (assuming these are present in this culture system).

8) GSK3B: The pY216 active form increases in spines (measured as ratio to total GSK3B) and inhibitors prevent spine retraction. But I don’t follow the logic that this inhibition therefore predicts that GSK3B would auto-phosphorylate itself, as stated in the results section: "We expected that changes in the phosphorylation state of GSK3β would occur by autophosphorylation of the Y216 activating site, since GSK3β inhibitors prevented dendritic spine retraction after 24 hrs of PrPSc treatment". If inhibitors are used, does phosphorylation no longer occur?

**********

PLOS authors have the option to publish the peer review history of their article (what does this mean?). If published, this will include your full peer review and any attached files.

If you choose “no”, your identity will remain anonymous but your review may still be made public.

**Do you want your identity to be public for this peer review?**  For information about this choice, including consent withdrawal, please see our Privacy Policy.

Reviewer #1: No

Reviewer #2: No

Reviewer #3: No

[NOTE: If reviewer comments were submitted as an attachment file, they will be attached to this email and accessible via the submission site. Please log into your account, locate the manuscript record, and check for the action link "View Attachments". If this link does not appear, there are no attachment files.]

**Figure resubmission:**

-->While revising your submission, we strongly recommend that you use PLOS’s NAAS tool (https://ngplosjournals.pagemajik.ai/artanalysis) to test your figure files. NAAS can convert your figure files to the TIFF file type and meet basic requirements (such as print size, resolution), or provide you with a report on issues that do not meet our requirements and that NAAS cannot fix.

After uploading your figures to PLOS’s NAAS tool - https://ngplosjournals.pagemajik.ai/artanalysis, NAAS will process the files provided and display the results in the "Uploaded Files" section of the page as the processing is complete. If the uploaded figures meet our requirements (or NAAS is able to fix the files to meet our requirements), the figure will be marked as "fixed" above. If NAAS is unable to fix the files, a red "failed" label will appear above. When NAAS has confirmed that the figure files meet our requirements, please download the file via the download option, and include these NAAS processed figure files when submitting your revised manuscript.

**Reproducibility:**

To enhance the reproducibility of your results, we recommend that authors of applicable studies deposit laboratory protocols in protocols.io, where a protocol can be assigned its own identifier (DOI) such that it can be cited independently in the future. Additionally, PLOS ONE offers an option to publish peer-reviewed clinical study protocols. Read more information on sharing protocols at https://plos.org/protocols?utm_medium=editorial-email&utm_source=authorletters&utm_campaign=protocols

# Author comment: Author response to Decision Letter 1

**DOI:** 10.1371/journal.ppat.1014314.r002

1 May 2026

Attachment Submitted filename: PLOS Patho 2026 rebutal final.docx

# Peer review: Decision Letter 1

**DOI:** 10.1371/journal.ppat.1014314.r003

**Authors:** Surachai Supattapone

26 May 2026

Dear %TITLE% Harris,

We are pleased to inform you that your manuscript 'Chemo-omic pipeline enables discovery of prion synaptotoxic pathways and inhibitory drugs' has been provisionally accepted for publication in PLOS Pathogens.

Before your manuscript can be formally accepted you will need to complete some formatting changes, which you will receive in a follow up email. A member of our team will be in touch with a set of requests.

Please note that your manuscript will not be scheduled for publication until you have made the required changes, so a swift response is appreciated.

IMPORTANT: The editorial review process is now complete. PLOS will only permit corrections to spelling, formatting or significant scientific errors from this point onwards. Requests for major changes, or any which affect the scientific understanding of your work, will cause delays to the publication date of your manuscript.

Should you, your institution's press office or the journal office choose to press release your paper, you will automatically be opted out of early publication. We ask that you notify us now if you or your institution is planning to press release the article. All press must be co-ordinated with PLOS.

Thank you again for supporting Open Access publishing; we are looking forward to publishing your work in PLOS Pathogens.

Best regards,

Suzette A. Priola

Academic Editor

PLOS Pathogens

Surachai Supattapone

Section Editor

PLOS Pathogens

Sumita Bhaduri-McIntosh

Editor-in-Chief

PLOS Pathogens

orcid.org/0000-0003-2946-9497

Michael Malim

Editor-in-Chief

PLOS Pathogens

orcid.org/0000-0002-7699-2064

***********************************************************

Reviewer Comments (if any, and for reference):

Reviewer's Responses to Questions

**Part I - Summary**

Please use this section to discuss strengths/weaknesses of study, novelty/significance, general execution and scholarship.

Reviewer #1: The authors now specify the methodology used to assess compound toxicity in their screen and included an additional figure (Figure S7) illustrating the morphology of collapsed actin bundles in neuronal cultures following Cymarin treatment. They further provided primary data quantifying spine numbers in mock and PrPSc-treated cultures in response to the tested compounds (Supplementary Table 7), which also clarifies their quantification approach. In addition, the methods section now states that image acquisition and analysis was conduced in a blinded and randomized manner. I find these additions satisfactory.

Reviewer #2: The authors have satisfactorily addressed all my previous concerns that were all very minor. No more concerns.

Reviewer #3: The authors have addressed by previous concerns.

**********

**Part II – Major Issues: Key Experiments Required for Acceptance**

Please use this section to detail the key new experiments or modifications of existing experiments that should be absolutely required to validate study conclusions.

Generally, there should be no more than 3 such required experiments or major modifications for a "Major Revision" recommendation. If more than 3 experiments are necessary to validate the study conclusions, then you are encouraged to recommend "Reject".

Reviewer #1: (No Response)

Reviewer #2: none

Reviewer #3: (No Response)

**********

**Part III – Minor Issues: Editorial and Data Presentation Modifications**

Please use this section for editorial suggestions as well as relatively minor modifications of existing data that would enhance clarity.

Reviewer #1: (No Response)

Reviewer #2: none

Reviewer #3: (No Response)

**********

PLOS authors have the option to publish the peer review history of their article (what does this mean?). If published, this will include your full peer review and any attached files.

If you choose “no”, your identity will remain anonymous but your review may still be made public.

**Do you want your identity to be public for this peer review?**  For information about this choice, including consent withdrawal, please see our Privacy Policy.

Reviewer #1: No

Reviewer #2: No

Reviewer #3: **Yes:** Valerie L Sim

# Editor report: Acceptance letter

**DOI:** 10.1371/journal.ppat.1014314.r004

**Authors:** Surachai Supattapone

Dear %TITLE% Harris,

We are delighted to inform you that your manuscript, "Chemo-omic pipeline enables discovery of prion synaptotoxic pathways and inhibitory drugs," has been formally accepted for publication in PLOS Pathogens.

We have now passed your article onto the PLOS Production Department who will complete the rest of the pre-publication process. All authors will receive a confirmation email upon publication.

The corresponding author will soon be receiving a typeset proof for review, to ensure errors have not been introduced during production. Please review the PDF proof of your manuscript carefully, as this is the last chance to correct any scientific or type-setting errors. Please note that major changes, or those which affect the scientific understanding of the work, will likely cause delays to the publication date of your manuscript. Note: Proofs for Front Matter articles (Pearls, Reviews, Opinions, etc...) are generated on a different schedule and may not be made available as quickly.

Soon after your final files are uploaded, the early version of your manuscript, if you opted to have an early version of your article, will be published online. The date of the early version will be your article's publication date. The final article will be published to the same URL, and all versions of the paper will be accessible to readers.

For Research Articles, you will receive an invoice from PLOS for your publication fee after your manuscript has reached the completed accept phase. If you receive an email requesting payment before acceptance or for any other service, this may be a phishing scheme. Learn how to identify phishing emails and protect your accounts at https://explore.plos.org/phishing.

Thank you again for supporting open-access publishing; we are looking forward to publishing your work in PLOS Pathogens.

Best regards,

Sumita Bhaduri-McIntosh

Editor-in-Chief

PLOS Pathogens

orcid.org/0000-0003-2946-9497

Michael Malim

Editor-in-Chief

PLOS Pathogens

orcid.org/0000-0002-7699-2064
